# The Antitumoral Effect of Paris Saponin II on Head and Neck Squamous Cell Carcinomas Mediated *via* the Nitric Oxide Metabolic Pathway

**DOI:** 10.3389/fcell.2021.803981

**Published:** 2022-01-03

**Authors:** Wenwen Qi, Fangyuan Zhu, Min Wang, Zhenxiao Teng, Runtong Xu, Yue Xi, Qiu Meng, Xinhao Wu, Hui Zhao, Min Ma, Xiaozhi Hou, Baowei Wang, Xiaoming Li, Chengcheng Liu, Xiang Zhang, Fenglei Xu, Ming Xia

**Affiliations:** ^1^ Department of Otolaryngology, Shandong Provincial Hospital, Cheeloo College of Medicine, Shandong University, Jinan, China; ^2^ Department of Otolaryngology, Shandong Provincial Hospital Affiliated to Shandong First Medical University, Jinan, China; ^3^ Department of Pathology, Shandong Provincial Hospital Affiliated to Shandong First Medical University, Jinan, China; ^4^ Cancer Center, Shandong Provincial Hospital Affiliated to Shandong First Medical University, Jinan, China; ^5^ Shandong Provincial Hospital Affiliated to Shandong First Medical University, Jinan, China; ^6^ Department of Pharmacy, Central Hospital Affiliated to Shandong First Medical University, Jinan, China

**Keywords:** Paris saponin II, head and neck squamous carcinomas, type III nitric oxide synthase, nitric oxide metabolic pathway, cancer stem cells

## Abstract

Paris saponin has shown great therapeutic value in cancer therapy. We used isolated Paris saponin II (PSII), an active component of Paris saponin, and demonstrated its antitumor effect on human head and neck squamous cell carcinoma cell lines. Additionally, we investigated its mechanisms of action *in vivo* by establishing a xenograft mouse model. The results showed that PSII had presented strong anticancer effects on both hypopharyngeal malignant tumor cell lines (FaDu) and laryngeal carcinoma cell lines (Tu212 and Tu686). In addition, we successfully isolated and cultured the head and neck squamous stem cells and the primary fibroblasts to perform metabonomics studies. The results showed that RPII remarkably decreased energy metabolism, and type III nitric oxide synthase 3 (NOS3) may be a target to block tumor growth. Furthermore, we found that PSII inhibited HNSCC proliferation and metastasis by inhibiting the nitric oxide metabolic pathway. Overall, these results demonstrated that PSII is a potent anticancer agent, and the metabonomics analysis is a valuable tool to investigate and establish the antitumor effects of traditional Chinese medicines.

## Introduction

Head and neck squamous cell carcinoma is one of the most common cancers in the world (Schinke et al., 2021), and its 5-year overall survival (OS) rate is only 45% (HammermanHammerman et al., 2015; Johnson et al., 2020). A possible reason for the failure of the cancer treatment is associated with the existence of “cancer stem cells” (CSCs) in tumors, which numerous studies have shown to have a major impact on the recurrence and metastasis of tumors, as well as the resistance to radio/chemotherapy treatment (Chen et al., 2020; Chang et al., 2021). Another possible reason is the tumor microenvironment (TME), which may have important implications for tumor therapy (Hinshaw and Shevde, 2019). Tumorigenesis is often driven by the changes in the living environment, which is referred to as TME (Wu and Dai, 2017). In the TME, the metabolites play an important role in several steps of tumorigenesis, including immune escape, local drug resistance, distant metastasis, and recurrence (Reina-Campos et al., 2017; García-Cañaveras et al., 2019; Li et al., 2019). In the past few decades, researchers have found a number of tumor suppressors and oncogenes, which are involved in the development of tumors, including some key metabolic enzymes, especially related to redox reactions and glycolysis (Jin et al., 2019; Ferro et al., 2020). The accumulation of protein aggregates, autophagic stress, oxidative stress, and mitochondrial dysfunction are common in many pathological processes of tumors (Lin et al., 2019). As indicated by many studies, oxidative stress can contribute to protein aggregation (Tripathi et al., 2020). Furthermore, mitochondrial dysfunction can promote DNA damage and instability by producing excessive amounts of reactive oxygen species (ROS) (Saki and Prakash, 20172017; Luo et al., 2020). Consequently, mitochondrial dysfunction is considered a metabolic hallmark of cancer cells. Metabolomics is a novel and convenient approach to explore the toxicity and features of medicine (Hsu et al., 2016; Vitale et al., 2019; Battaglia et al., 2020). Therefore, metabonomics is a promising approach to investigate the mechanisms and safety of medicine.

Paridis saponins (PSs), a natural extract of Paris polyphylla Smith var. yunnanensis used as an anticancer drug in traditional Chinese medicine (TCM) which is also an active component of Paris saponin II (PSII), has shown a strong antitumor power in various cancers, such as hepatocellular carcinoma (HCC) (Cheng et al., 2008), lung cancer (Man et al., 2015), and ovarian cancer (Xiao et al., 2009). Recent studies have reported promising roles of PS in the regulation of glycolytic and lipid metabolism (Man et al., 2017; Man et al., 2020). All these results indicate that PSII may be a valuable anti-HNSCC agent. In this study, we used a metabonomics approach to investigate the metabolic features in the HNSCC cell lines treated with PSII. The marker metabolites were used to evaluate the anticancer capacity of drugs (Sak and Everaus, 2015). After analyzing the changes in these metabolomics in PSII-treated HNSCC cell lines by a metabonomics approach, we investigated the mechanism mediating the antitumor effects of PSII intervening tumor development. In addition, a mouse model was developed to explore the inhibitory effect of PSII on HNSCC growth (Supsavhad et al., 2016; Gilardi et al., 2020). Our study demonstrated that PSII is a potent anticancer agent in the HNSCC. Additionally, it also showed that a metabonomics approach can be a valuable tool to elucidate the antitumor effects of TCM preparations.

## Materials and Methods

### Materials

We purchased Paris saponin II (PSII) (purity > 99%) from Chengdu Must Bio-technology Technology Co., Ltd (Chengdu, China). The HNSCC cell lines including the hypopharyngeal cancer cell line FaDu and the laryngeal carcinoma cell lines Tu212 and Tu686 were purchased from American Type Culture Collection (ATCC; Rockville, MD, United States). Antibodies against Bcl-2, Ki67, nitric oxide synthase 3 (NOS3), CytC, Lc3b, β-actin, and Bax were purchased from Cell Signaling Technology Inc. (CST Inc., Danvers, MA, United States).

### Cell Culture

Two cell lines (Tu686 and FaDu) were cultured in Dulbecco’s modified Eagle medium (DMEM) which were obtained from Thermo Fisher Scientific Inc./Gibco (Waltham, MA, United States) supplemented with 10% fetal bovine serum (FBS) obtained from Haoyang Biological Manufacture Co. Ltd. (Tianjin, China), as well as penicillin/streptomycin (P/S; 100 U/ml/100 pg/ml). Tu212 cell lines were cultured in Iscove’s Modified Dulbecco’s Medium (IMDM) which was bought from Thermo Fisher Scientific Inc./Gibco supplemented with 10% FBS and P/S. All the cell lines were routinely cultured in a humidified cell incubator at 37°C with 5% CO_2_.

### Cell Viability Assay

We measured cell viability using the methyl thiazolyl tetrazolium (MTT) assay. FaDu, Tu686, and Tu212 cells were plated in 96-well plates at a cell concentration of 3 × 10^3^ cells/well. After overnight incubation for one night, the cultured medium was changed with a new medium containing different concentrations of PSII (0, 0.025, 0.05, 0.1, 0.2, 0.4, 0.8, and 1.6 μg/ml), and then we incubated them for 24, 48, 72, and 96 h at 37°C with 5% CO_2_ in a cell incubator, Afterward, 20 µl of MTT reagent (5 mg/ml) was added to each well, and the cells were incubated for 4 h at 37°C. Subsequently, the supernatants in the wells were cautiously aspirated, and 100 µl of dimethyl sulfoxide (DMSO) solution was added to each well. The 96-well plates were shaken for 10 min at room temperature and then the absorbance values were measured at 570 nm on a microplate reader (Bio-Rad Laboratories Inc., Hercules, CA, United States).

### Invasion and Migration Assays

We used transwell plates (Coming Costar, Lowell, MA, United States) to assay cell invasion and migration. In the migration assay, we starved the cells in a serum-free medium (SFM) for 12 h at 37°C with 5% CO_2_. We then added 700 µl of DMEM or IMDM with 20% FBS to the lower well and 500 µl of SFM, including 1 × 10^5^ cells to the upper transwell inserts. After culturing for 48 h at 37°C with 5% CO_2_, we counted the number of cells that adhered to the lower surface of the insert membrane. We performed the invasion assay in the same way, except that the transwell insert membrane was coated with Matrigel (BD Biosciences, San Jose, CA, United States).

### Colony Forming Assay

We seeded the three HNSCC cell lines in six-well plates at a cell concentration of 1 × 10^3^ cells/well, and the plates were incubated for 24 h at 37°C with 5% CO_2_. After incubation for 24 h, we replaced the old medium with a new medium containing PSII and continued incubation for 10 days. Subsequently, after washing cells with phosphate-buffered saline (PBS), the cells were fixed with methyl alcohol and stained with a 1% crystal violet solution. Ultimately, after washing the cells with PBS, we observed the cells under a microscope and acquired images of various fields to count the number of colonies.

### Cell Scratch Test Assay

Once cultured, the cells reached close to 100% confluence, the cell monolayer was mechanically scratched. Scratch healing was observed at 0, 12, 24, and 48 h under a microscope at ×40 magnification (white light bright field). We used ImageJ software [National Institute of Health (NIH), Bethesda, MD, United States] to calculate the migration area on the scratch, using the following formula: Scratch area rate (%) = 12, 24, and 48 h after migration scratch area/initial scratch area ×100%.

### Cell Line Microsphere Culture for Stem Cell

The corresponding SFM was prepared by adding double antibody as follows: 20 ng/ml epidermal growth factor (EGF; PeproTech US, Cranbury, NJ, United States), 20 ng/ml basic fibroblast growth factor (bFGF, Thermo Fisher Scientific Inc./Gibco), and 2% B27 (Invitrogen, Carlsbad, CA, United States) were added into DMEM/F12 culture medium (Thermo Fisher Scientific Inc./Gibco). Logarithmic growth phase HNSCC cell lines (FaDu, Tu212, and Tu686) were digested with 0.25% trypsin (Thermo Fisher Scientific Inc./Gibco) and dissociated into a single cell suspension. Then the resuspended cells were inoculated into SFM at a density less than 10^4^/ml for routine culture and added into an ultralow adsorption T25 or smaller glass flask, which was placed vertically in an incubator and shaken several times a day. The formation of microspheres was observed, and half of the cell suspension solution was replaced every 2–3 days, and the cells were subcultured every 6–8 days. During subculturing, microspheres were collected by centrifugation and resuspended in an accutase solution (MilliporeSigma, Burlington, MA, United States) and digested at 37°C for 5–10 min. The microspheres were pipetted up and down several times until they dispersed into single cells. After centrifugation and washing, the microspheres were counted and inoculated into SFM for subculture at a density less than 10^4^/ml.

### Flow Cytometry

Logarithmic growth phase tumor cells and stem cells were collected, digested, centrifuged, and resuspended in PBS. After counting, the number of cells was adjusted to 1 × 10^6^ cells/group. Anti-CD24-fluorescein isothiocyanate (FITC) was used as negative control, and the proportion of anti-CD44-phycoerythrin (PE)/anti-CD133-allophycocyanin (APC) cells in each group was determined by flow cytometry analysis. When the anti-CD44-PE and anti-CD133-APC were both positive, it revealed the presence of stem cells.

### Primary Culture of Fibroblasts From Head and Neck Tumor Tissue

We selected the pathology of patients with hypopharyngeal malignant tumor for the first time as experimental subjects which was approved by the Ethics Committee at our institution. When rapid intraoperative pathology confirmed that the pathological type was squamous cell carcinoma of the head and neck, we collected tumor tissues for study. The head and neck tumor tissues were washed three times with cold PBS mixed with double antibodies in an ultraclean workbench. Then the tissue blocks were placed in a 10-cm sterile petri dish and cut into 1- to 2-mm tissue blocks with cold PBS as described to wash away the free cells. Subsequently, using tweezers, the tissue blocks were placed on the bottom surface of a culture flask with a spacing of about 0.5 cm. The flask was inverted, and 3 ml of the medium (DMEM+20%FBS + double antibody) was added to the bottom of the flask, and the tissue blocks were incubated for 4 h. Then 3 ml of the medium was added again, and the culture flask was inverted and incubated for an additional 48 h. The growth of primary tumor–associated fibroblasts was observed under a microscope, and the migration ability of the purified cells was examined by a transwell migration assay.

### Immunofluorescence for Identifying Primary Culture of Fibroblasts

Tumor-associated fibroblasts were seeded into a petri dish with a pretreated cover glass and allowed to grow. After the cells had nearly formed a monolayer, the cover glass was removed, and the cells were washed twice with PBS. Then cells were fixed for 2 h with paraformaldehyde, permeabilized with 5% Triton X-100 for 20 min, blocked with 2% BSA, and washed twice with PBS. The cells were treated with the corresponding primary antibody against FAP (CST Inc.) and SMA (Abcam, Cambridge, United Kingdom), followed by incubation with appropriate secondary antibodies. Ultimately, all fibroblasts were stained with 4’,6-diamidino-2-phenylindole (DAPI).

### High-Resolution Untargeted Metabolomics Analysis

We performed untargeted metabolomics analysis of the three HNSCC cell lines treated with/without PSII. The metabolites in the samples were analyzed by ultrahigh performance liquid chromatography–quadrupole time-of-flight mass spectrometry (UHPLC-Q-TOF MS). The retention time and molecular weight of the metabolites in the samples were compared with those in the local database. Also, the 25 PPM, secondary fragmentation spectrum, collision energy, and other information were matched to identify the structure of metabolites in biological samples, and the identification results were confirmed through strict manual secondary check and confirmation. The assessment level is Level 2.

### Western Blot Analysis

FaDu and Tu686 cells were treated with PSII (0, 0.05, and 0.1 μg/ml) for 48 h at 37°C with 5% CO_2_. Cells were collected and lysed in lysis buffer with protease inhibitors to extract proteins. We separated proteins by 12% sodium dodecyl sulfate–polyacrylamide gel electrophoresis (SDS-PAGE). Then the separated proteins were electroblotted onto a polyvinylidene difluoride (PVDF) membrane using a blotting apparatus (Bio-Rad Laboratories). After blocking the membranes with 5% non-fat milk, the membranes were incubated overnight at 4°C with the corresponding primary antibodies, followed by labeling with the appropriate secondary antibody at room temperature for 1 h. We used β-actin as the internal control. Eventually, the immunoreacted protein bands were visualized using the Pro-lighting horseradish peroxidase (HRP) agent for Western blotting detection (Tiangen Biotech Co., Ltd., Beijing, China). The Western blot analysis of mouse xenograft tumors was performed in the same way as that for the cells.

### Quantitative Real-Time Polymerase Chain Reaction (qRT-PCR) Analysis

Total RNA was isolated from FaDu and Tu686 cells using TRIzol (Invitrogen), and the quality of the isolated RNA by the absorbance at 260 and 280 nm was determined. cDNA synthesis was carried out using Rever-tAidTM M-MuLV RT (Fermentas, Hanover, MD, United States) according to the instructions, and the obtained cDNA was stored at −80°C. Polymerase chain reactions were performed in a final volume of 50 ml including 2 µl each of the forward and reverse primers, 25 µl 2*Taq Master Mix, 2 µl cDNA, and RNase-free water. After transfection, total RNA was obtained from the transfected cells with TRIzol, and cDNA was obtained by reverse transcription as described. Real-time quantitative PCR was performed to detect the mRNA level.

### Transfection of Cells

The primers used were synthesized by Ding Guo Changsheng Biotechnology Co., Ltd. (Beijing, China). The NOS3-F primer sequence is GTG​ATG​GCG​AAG​CGA​GTG​AAG​G, and the NOS3-R primer sequence is CAC​CAC​GTC​ATA​CTC​ATC​CAT​ACA​CAG. The FaDu and Tu686 cells were inoculated in six-well plates, and when confluence reached about 85%, siRNA targeting NOS3 (NOS3-siRNA) was diluted with serum-free DMEM and mixed gently with Lipofectamine 2000 (Biyuntian Biological Technology Co., Shanghai, China). After mixing, the mixture was incubated for 20 min at room temperature. Afterward, FaDu and Tu686 cells were plated in culture plates to transfect for 6 h and then cultured in a complete medium for 48 h.

### Xenograft Tumorigenicity Assay

A total of 5 × 10^6^ cells in 0.2 ml of PBS were injected subcutaneously into 4-week-old BALB/c nude female mice purchased from Taconic Biosciences (Rensselaer, NY, United States) After 5 days, the mice were randomly assigned to two groups, namely, the PSII group and control group. The PSII group was administered with about 100 µl of solution (5 mg/kg) by intraperitoneal injection once every 3 days. We measured the tumor growth once every 5 days according to the following equation: Volume = (width^2^ × length)/2. On the 60th day, mice were anesthetized with ether and then killed. Eventually, the xenograft tumors were removed and processed for immunohistochemical (IHC) analysis.

### Histopathological Examination

The spleen, lung, liver, heart, and kidney tissues of the mice were fixed in 10% formalin for the histopathological examination. After the dehydration process, we embedded the tissues in paraffin wax and cut them into 5-µm-thick sections using microtome. Then we stained the sections with hematoxylin and eosin (H&E). Ultimately, H&E-stained histopathology images were acquired using an Olympus microscope (Olympus Corporation, Tokyo, Japan).

### Immunohistochemical (IHC) Analysis

First, we cut the xenografts into 5-µm-thick sections using microtome. Then the slices were labeled and placed in an oven at 60°C for 2 h. After dewaxing the paraffin sections, the endogenous peroxidases of the sections were blocked. Subsequently, the sections were boiled in 10 mM sodium citrate plus 0.05% Tween-20 (pH 6) for 10 min in a microwave oven for antigen retrieval. The tissue sections were uniformly covered with 5% BSA and sealed at 37°C for 30 min. Then the sections were incubated with the corresponding primary antibody for 12 h at 4°C in the dark. Afterward, the sections were washed three times with PBS and incubated with the appropriate secondary antibody (1:200) for 60 min at room temperature. Then the sections were stained using a diaminobenzidine (DAB) HRP Color Development Kit (Beyotime Biotechnology, Shanghai, China) according to the manufacturer’s instructions. Next, the sections were stained with hematoxylin to stain the nucleus, dehydrated, and covered with neutral resin. Eventually, images of the sections were acquired on an Olympus microscope.

### Statistical Analysis

SPSS (version 20.0) software (IBM Corporation, Armonk, NY, United States) was used for statistical analysis. One-way analysis of variance (ANOVA) and Student’s *t*-test were performed to evaluate the significance. Every test was performed three times, and the error bars represent the standard error of the mean (SEM) in which differences with *p* < 0.05 were considered significant.

## Results

### Preparation and Characterization of Paris Saponin II

**FIGURE 1:**
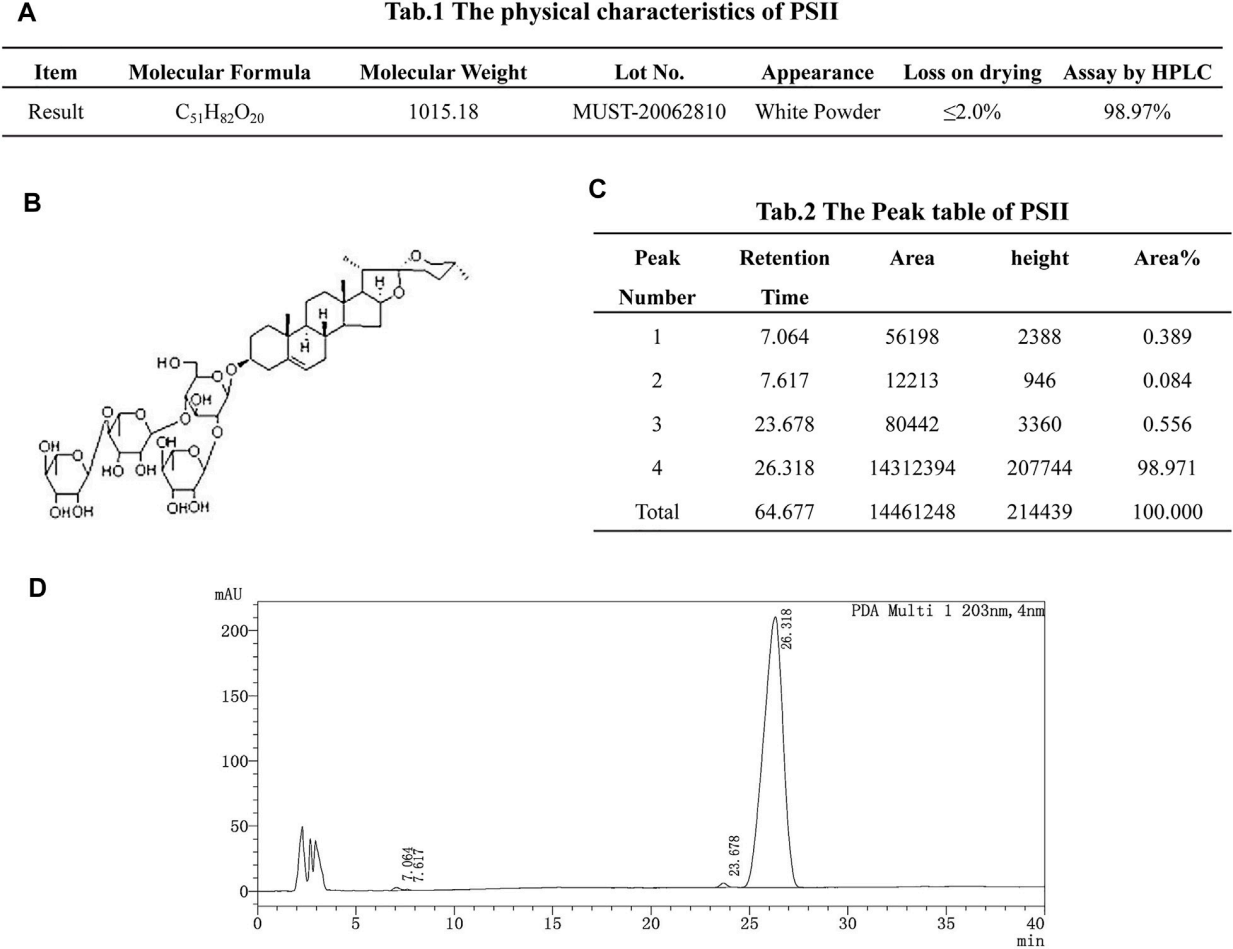
Drug instruction. **(A)** The physical characteristics of PSII. **(B)** Structural formula of PSII. **(C)** The peak table of PSII. **(D)** The peak figure of PSII. Eluant: acetonitrile:water = 50/50, 50 min. Flow velocity: 1.0 ml/min. Chromatographic column: MinXi Teeh CG-C18 5mic 250 × 4.6 mm. Column temperature: 40°C. The sample solvent: methyl alcohol.

As shown in Figure 1A, the molecular formula of PSII is C51H82O20, and its molecular weight is 1015.18. PSII has a white powder appearance, and its structural formula is as shown in Figure 1B. Chromatographic separation at a column temperature of 40°C, using a MinXi Teeh CG-C18 5 µm 250 × 4.6-mm chromatography column, with methyl alcohol as sample solvent, resulted in four peaks.

### PSII Inhibited the Proliferation of HNSCC Cell Lines

**FIGURE 2:**
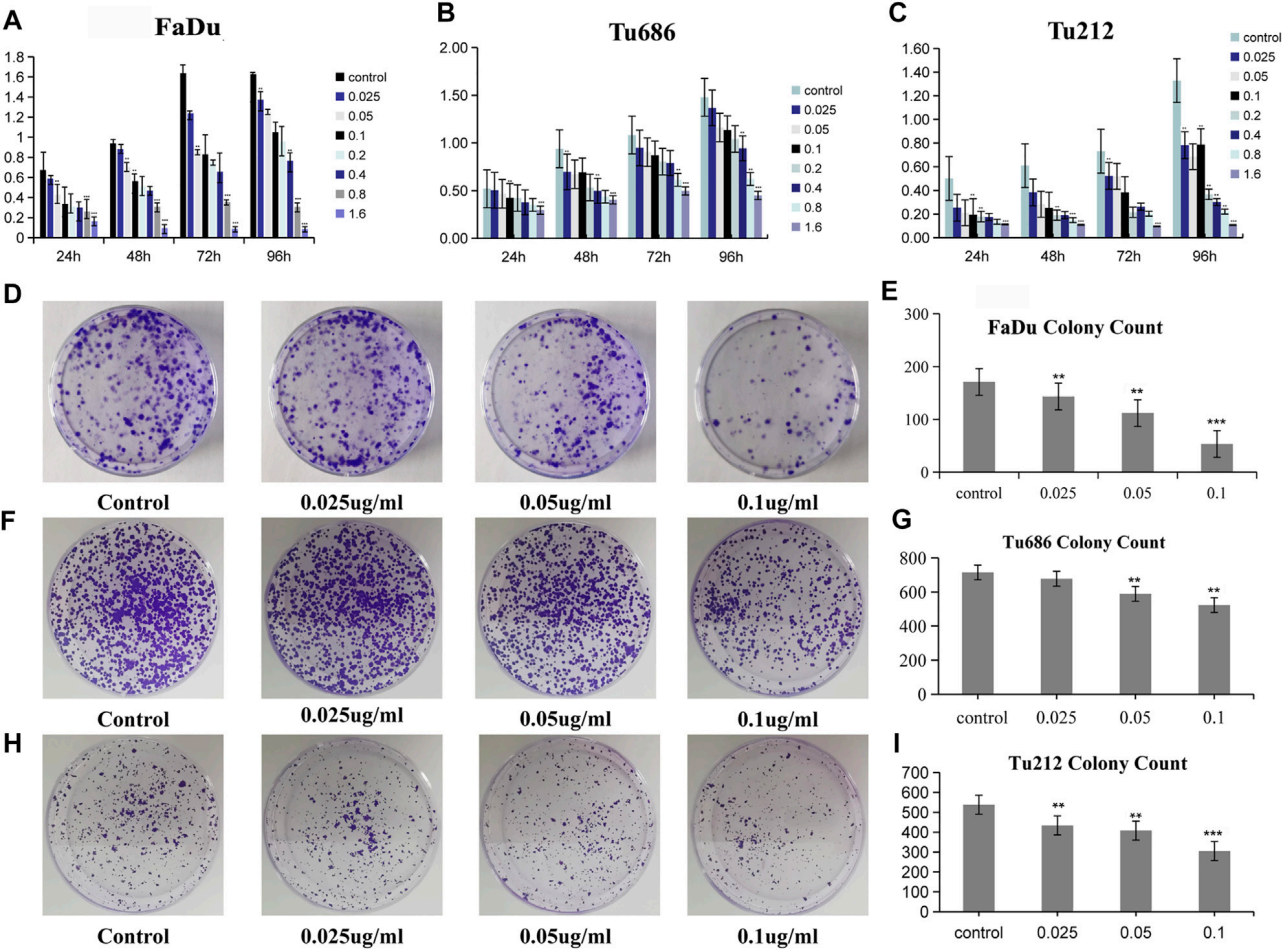
PSII reduced the viability of HNSCC cell. **(A)** The inhibitory effect of PSII on FaDu cell viability by MTT. **(B)** The inhibitory effect of PSII on Tu686 cell viability by MTT. **(C)** The inhibitory effect of PSII on Tu212 cell viability by MTT. **(D)** FaDu cells formed colonies. **(E)** The statistical figure of FaDu cells clone formation assay. **(F)** Tu686 cells formed colonies. **(G)** The statistical figure of Tu686 clone formation assay. **(H)** Tu212 cells formed colonies. **(I)** The statistical figure of Tu212 clone formation assay. Bars, 50 µm. All the experiments were carried out three times. The bar graphs and the table show quantification of the results, and each data represent the mean ± SD of three independent experiments (*p < 0.05; **p < 0.01; ***p < 0.001 vs the control group).

The measurement of cell proliferation by the MTT assay, as shown in Figures 2A–C, revealed that treatment with PSII significantly inhibited the proliferation of FaDu, Tu686, and Tu212 cells in a time- and concentration-dependent manner, especially after 48 h or at a concentration of 0.1 μg/ml PSII. In addition, the colony formation assay also indicated that PSII inhibited the proliferation of FaDu, Tu686, and Tu212 cells (Figures 2D–I).

### PSII Reduced the Motility of HNSCC Cells

**FIGURE 3:**
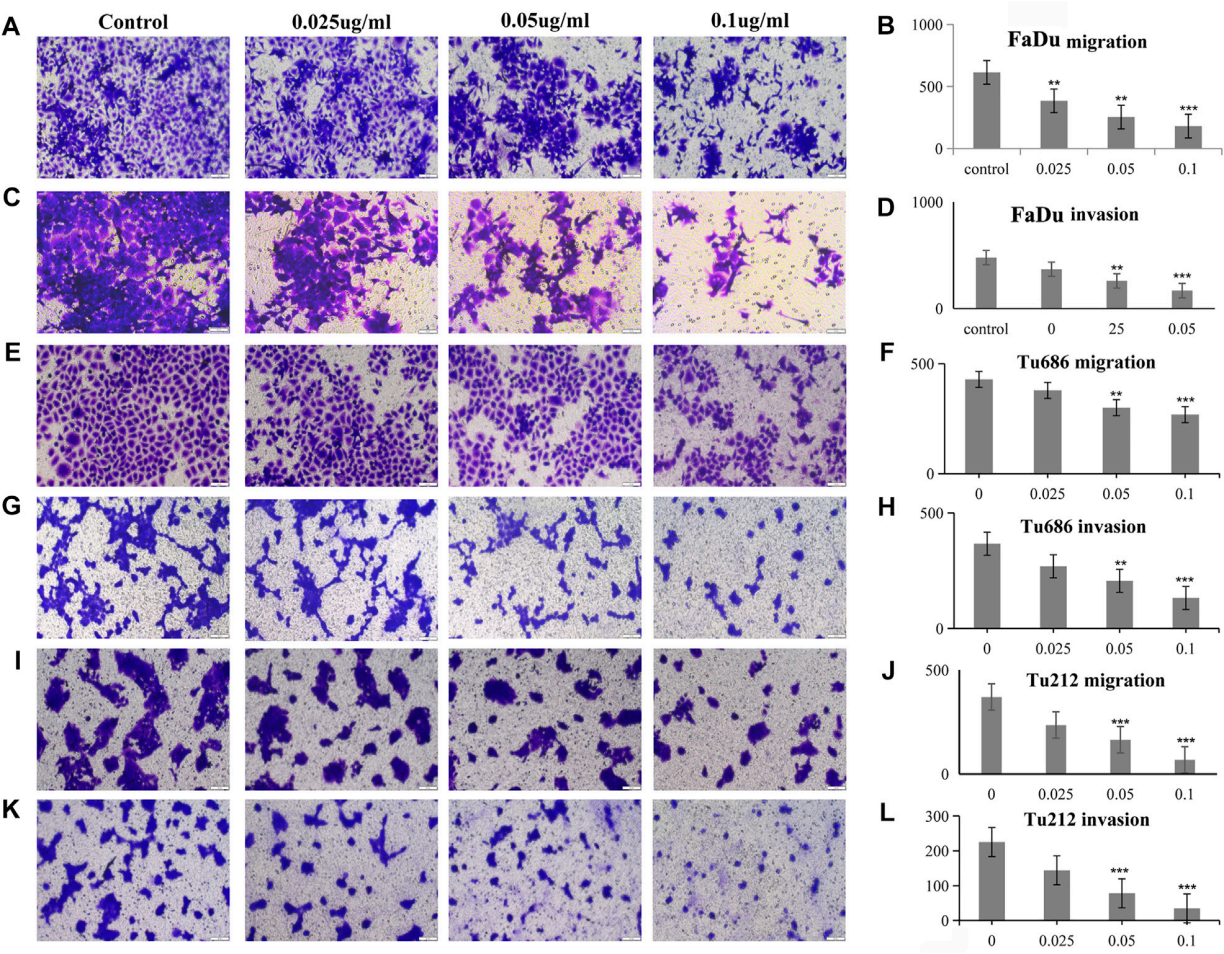
PSII reduced the motility of human squamous cell carcinoma cell by transwell assay. **(A)** PSII inhibited FaDu cell mobility by migration. **(B)** The statistical figure of FaDu cell migration assay. **(C)** PSII inhibited FaDu cell mobility by invasion. **(D)** The statistical figure of FaDu cell invasion assay. **(E)** PSII inhibited Tu686 cell mobility by migration. **(F)** The statistical figure of Tu686 cell migration assay. **(G)** PSII inhibited Tu686 cell mobility by invasion. **(H)** The statistical figure of Tu686 cell invasion assay. **(I)** PSII inhibited Tu212 cell mobility by migration. **(J)** The statistical figure of Tu212 cell migration assay. **(K)** PSII inhibited Tu212 cell mobility by invasion. **(L)** The statistical figure of Tu212 cells invasion assay. Bars, 50 µm. All the experiments were carried out three times. The bar graphs and the table show quantification of the results, and each data represent the mean ± SD of three independent experiments (*p < 0.05; **p < 0.01; ***p < 0.001 vs the control group).

**FIGURE 4:**
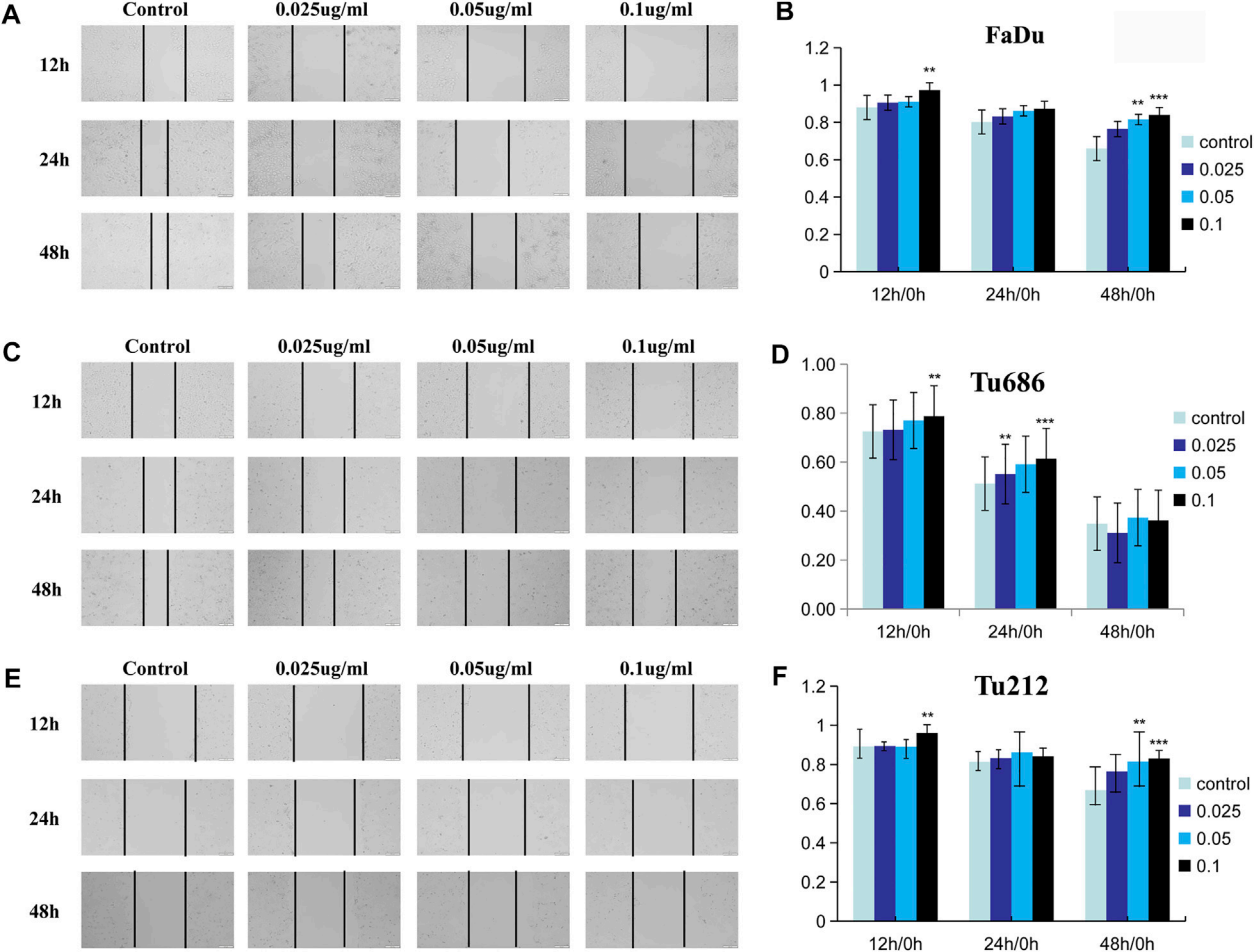
PSII reduced the motility of human squamous cell carcinoma cell by cell scratch test. **(A)** PSII inhibited FaDu mobility by the cell scratch test in a dose- and time-dependent manner. **(B)** The statistical figure of FaDu cells scratch test. **(C)** PSII inhibited Tu686 mobility by the cell scratch test in a dose- and time-dependent manner **(D)**. The statistical figure of Tu686 cells scratch test. **(E)** PSII inhibited Tu212 mobility by the cell scratch test in a dose- and time-dependent manner **(F)**. The statistical figure of Tu212 cell scratch test. Bars, 50 µm. All the experiments were carried out three times. The bar graphs and the table show quantification of the results, and each data represent the mean ± SD of three independent experiments (*p < 0.05; **p < 0.01; ***p < 0.001 vs the control group).

The analysis of tumor cell motility by transwell assays and cell scratch assays revealed, as shown in Figure 3, that the PSII extract inhibited migration and invasion in FaDu, Tu686, and Tu212 cells. In particular, the cell scratch assay results in Figure 4 showed the inhibitory effect of PSII on these three HNSCC cell lines, and cell motility clearly decreased after treatment with 0.1 μg/ml PSII.

### PSII Inhibited the Growth of HNSCC Microsphere Cells

**FIGURE 5:**
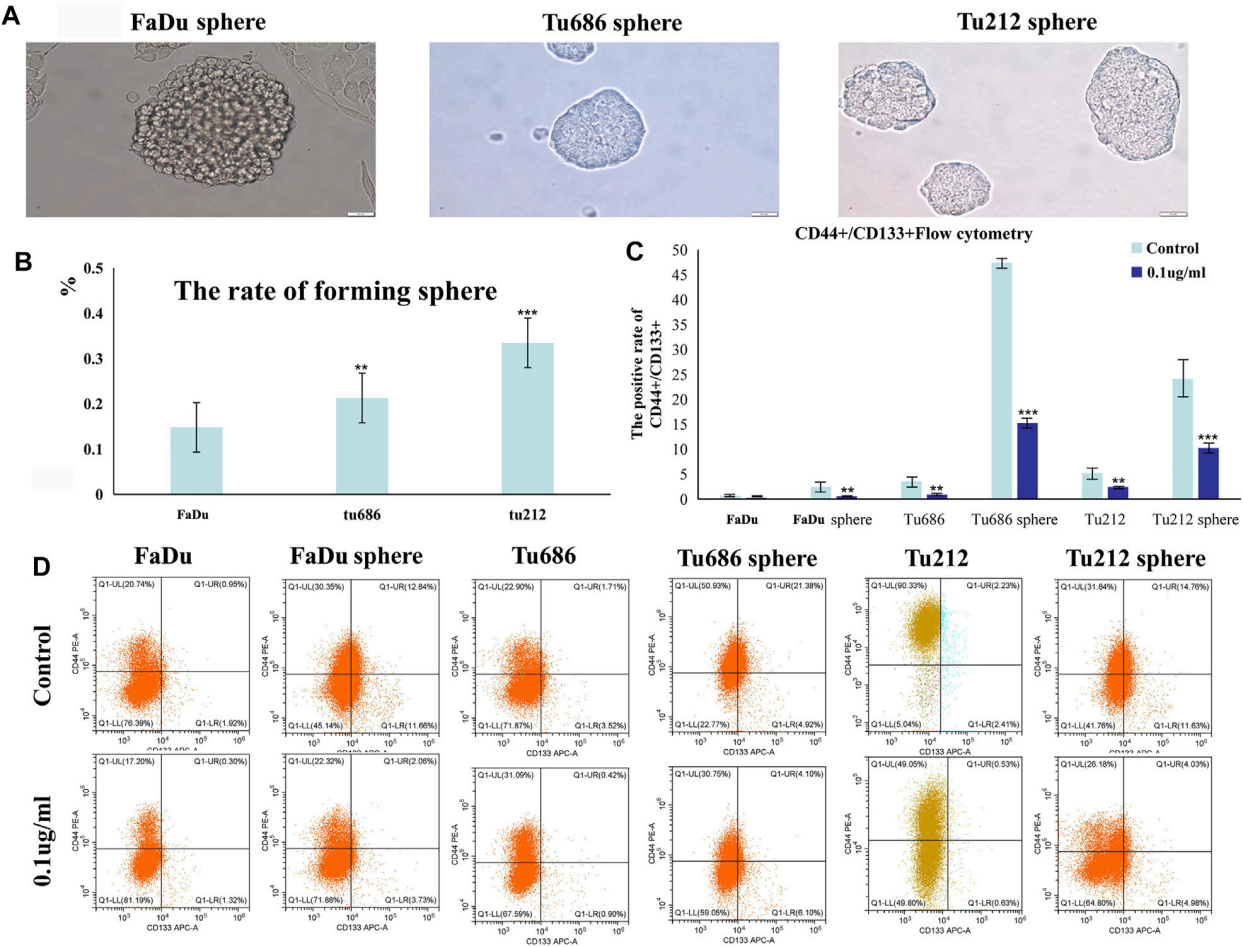
Growth of human squamous cell carcinoma microsphere cells cultured in the serum-free medium. **(A)** Human squamous cell carcinoma spheres generated in SFM. **(B)** The statistical figure of forming sphere rate. **(C)** The positive rate of CD44+/CD133 + by flow cytometry. **(D)** Representative flow cytometry pictures of the cell stem are shown. Bars, 50 µm. All the experiments were carried out three times. The bar graphs and the table show quantification of the results, and each data represent the mean ± SD of three independent experiments (*p < 0.05; **p < 0.01; ***p < 0.001 vs the control group).

We prepared SFM by adding 20 ng/ml bFGF, 20 ng/ml EGF, and 2% B27. After subculturing four times in this SFM, we calculated the pelletizing rate of tumor cells (≥100 μm), as shown in Figure 5B. Anti-CD24-FITC was used as a negative control, and the proportion of anti-CD44-PE/anti-CD133-APC cells in each group was determined by flow cytometry. The results shown in Figures 5C,D indicated that PSII suppressed the stemness of carcinoma cell stem microspheres.

### PSII Significantly Reduced the Viability of Primary Tumor–Associated Fibroblasts

**FIGURE 6:**
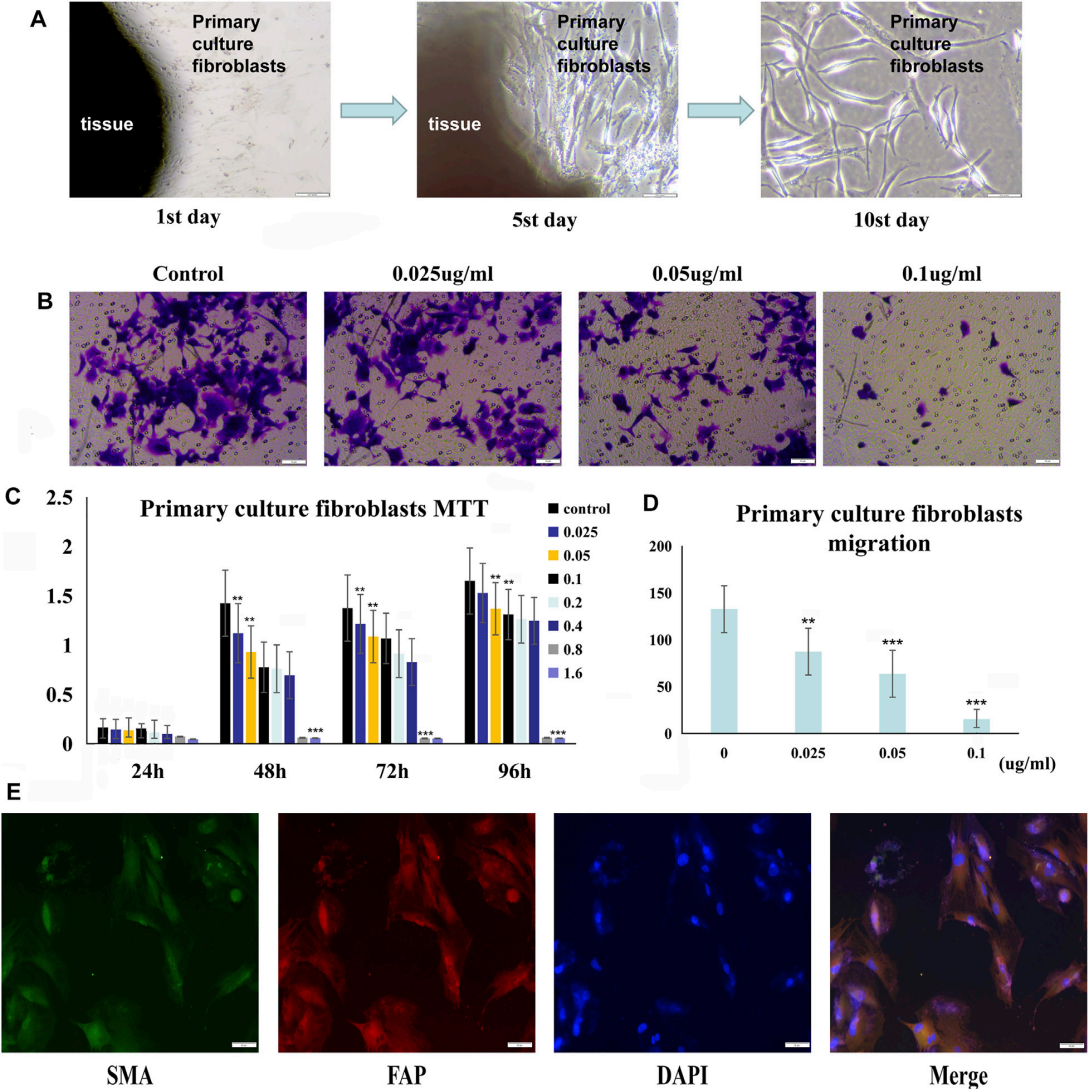
PSII reduced the growth and motility of primary culture fibroblasts from head and neck tumor tissue. **(A)** The process of human squamous cell carcinoma primary culture fibroblasts as described in Materials and Methods. **(B)** The migration of cancer primary culture fibroblasts in a dose-dependent manner. **(C)** The MTT statistical figure of cancer primary culture fibroblasts in a dose- and time-dependent manner. **(D)** The migration statistical figure of cancer primary culture fibroblasts in a dose-dependent manner. **(E)** Immunofluorescence for identifying primary culture of fibroblasts. Cells were treated with the corresponding primary antibody against FAP, SMA, followed by incubation with appropriate secondary antibodies and all fibroblasts were stained with DAPI. Bars, 50 µm. All the experiments were carried out three times. The bar graphs and the table show quantification of the results, and each data represent the mean ± SD of three independent experiments (*p < 0.05; **p < 0.01; ***p < 0.001 vs the control group).

After we successfully isolated primary tumor–associated fibroblasts, we used immunofluorescence analysis to phenotypically identify these cells. We measured the viability of the primary tumor–associated fibroblasts by the MTT assay. As shown in Figure 6C, treatment with PSII significantly reduced the viability of primary tumor–associated fibroblasts. In addition, the results of the migration assay shown in Figure 6D demonstrated the inhibitory effect of PSII on the migration ability of primary tumor–associated fibroblasts.

### PSII Had an Impact on the Metabolites in HNSCC Cell Lines

Principal component analysis (PCA) performed on the peaks extracted from all experimental samples revealed, as shown in Figures 10A,B, that the experiments had good repeatability, as indicated by their close clustering together in positive and negative ion modes. All the metabolite tests in this study (identified with positive and negative ions) were classified and counted according to the attribution information of their chemical taxonomy. The proportions of various metabolites are shown in Figures 10C,D. The histograms used to visually display the identified multiple changes of significantly differentially expressed metabolites, and multiple analyses of significantly differentially expressed metabolites in positive and negative ion modes are shown in Figures 10E,F. The abscissa in the histogram graphs represents the differential expression multiple, red color represents the differential expression multiple greater than 1, and the green color represents the differential expression multiple less than 1. The ordinate indicates the significantly different metabolites.

### NOS3 May Be a Downstream Target of PSII

**FIGURE 7:**
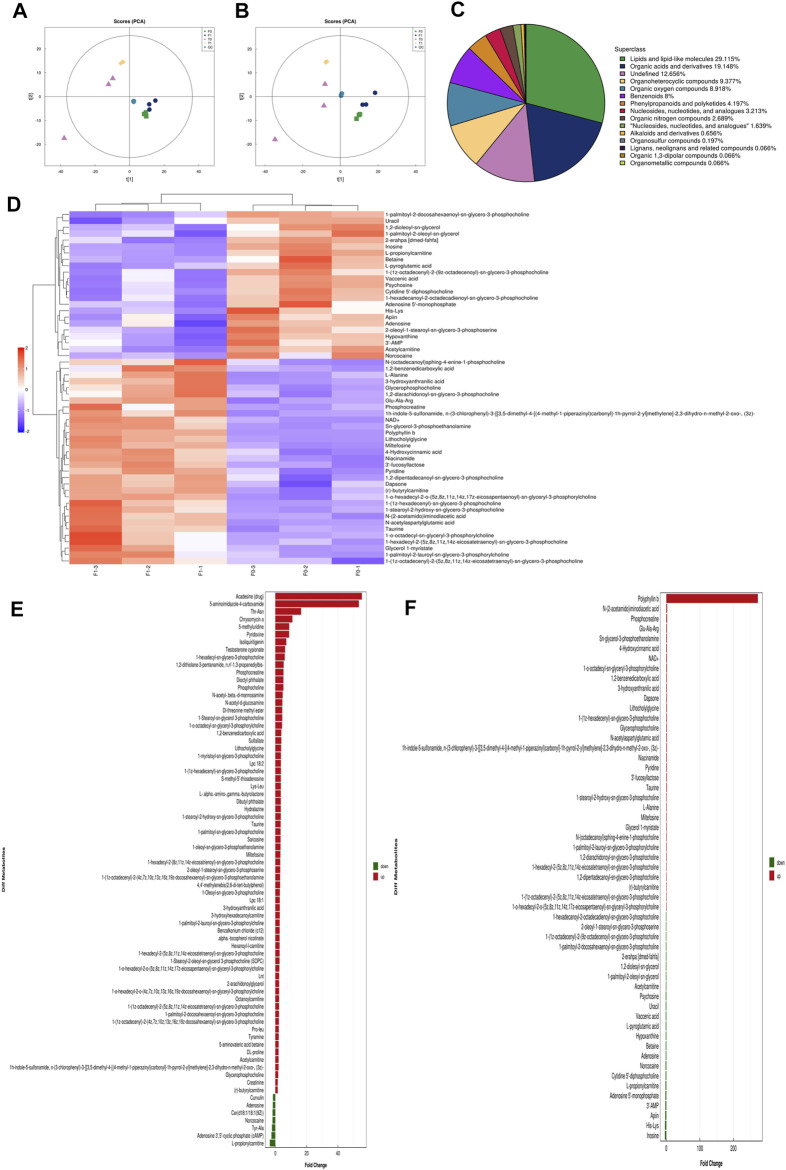
High-resolution untargeted metabolomics analysis. **(A)** PCA analysis diagram of positive ion model population sample. **(B)** PCA analysis diagram of the total sample of anion mode. **(C)** The proportion of the number of various metabolites. **(D)** Hierarchical cluster heat map of significant differential metabolites. **(E)** Histogram of the identified multiple changes of significant differential metabolites, and multiple analyses of significant differential metabolite expression in positive ion mode. **(F)** Histogram of the identified multiple changes of significant metabolic differences and multiple analyses of metabolite expression in negative ion mode. All the experiments were carried out three times. Each data represent the mean ± SD of three independent experiments (*p < 0.05; **p < 0.01; ***p < 0.001 vs the control group).

**FIGURE 8:**
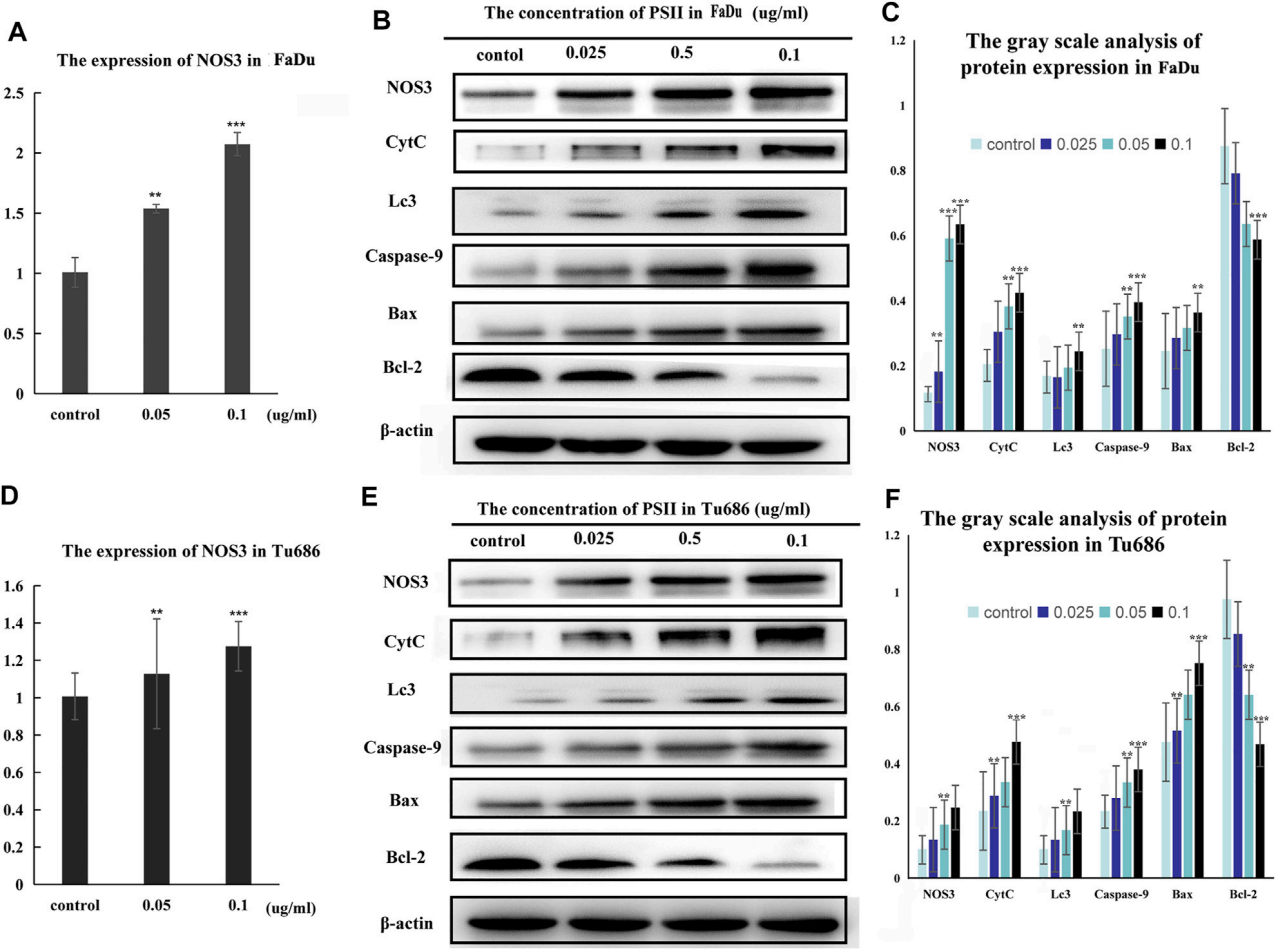
Signal pathway inducted by PSII in HNSCC cells. **(A)** The mRNA expression of NOS3 in FaDu cells by RT-PCR assay. **(B)** PSII treatment induced apoptosis and autophagy by mitochondrial pathway (NOS3, CytC, Lc3, caspase-9, Bax, and Bcl-2) in FaDu cells. **(C)** The gray scale analysis of protein expression in FaDu cells by Western blot. **(D)** The expression of NOS3 mRNA in Tu686 cells by RT-PCR assay. **(E)** PSII treatment induced apoptosis and autophagy by mitochondrial pathway (NOS3, CytC, Lc3, caspase-9, Bax, and Bcl-2) in Tu686 cells. **(F)** The gray scale analysis of protein expression in Tu686 cells by Western blot. We used β-actin as control. All the experiments were carried out three times. Each data represent the mean ± SD of three independent experiments (*p < 0.05; **p < 0.01; ***p < 0.001 vs the control group).

Treatment of FaDu and Tu686 cells with PSII (0, 0.025, 0.05, and 0.1 ug/ml) for 48 h revealed, as shown in Figure 7, that the mRNA and protein expression levels of genes involved in the mitochondrial pathway (CytC), cell autophagy (Lc3b), and apoptosis (Bax, Bcl-2, and caspase-9) were all altered by treatment with PSII. We used the Chinese Medicine Data website (http://herb.ac.cn/) to predict the target of PSII in cancer, and the result suggested many targets. Considering the metabolite results, we supposed that NOS3 may be a target. Additionally, NOS3 knockdown experiments using siRNA against NOS3 in HNSCC cells confirmed the effects of NOS3. Moreover, Western blot analysis confirmed that the protein expression levels of Bax, CytC, and Lc3b were decreased and that of Bcl-2 was increased when NOS3 expression was knocked down in FaDu and Tu686 cells (Figures 8C,D). These results confirmed that NOS3 is a downstream target of PSII in HNSCC.

### PSII Inhibited the Growth of Subcutaneous Tumors by Regulating NOS3

**FIGURE 9:**
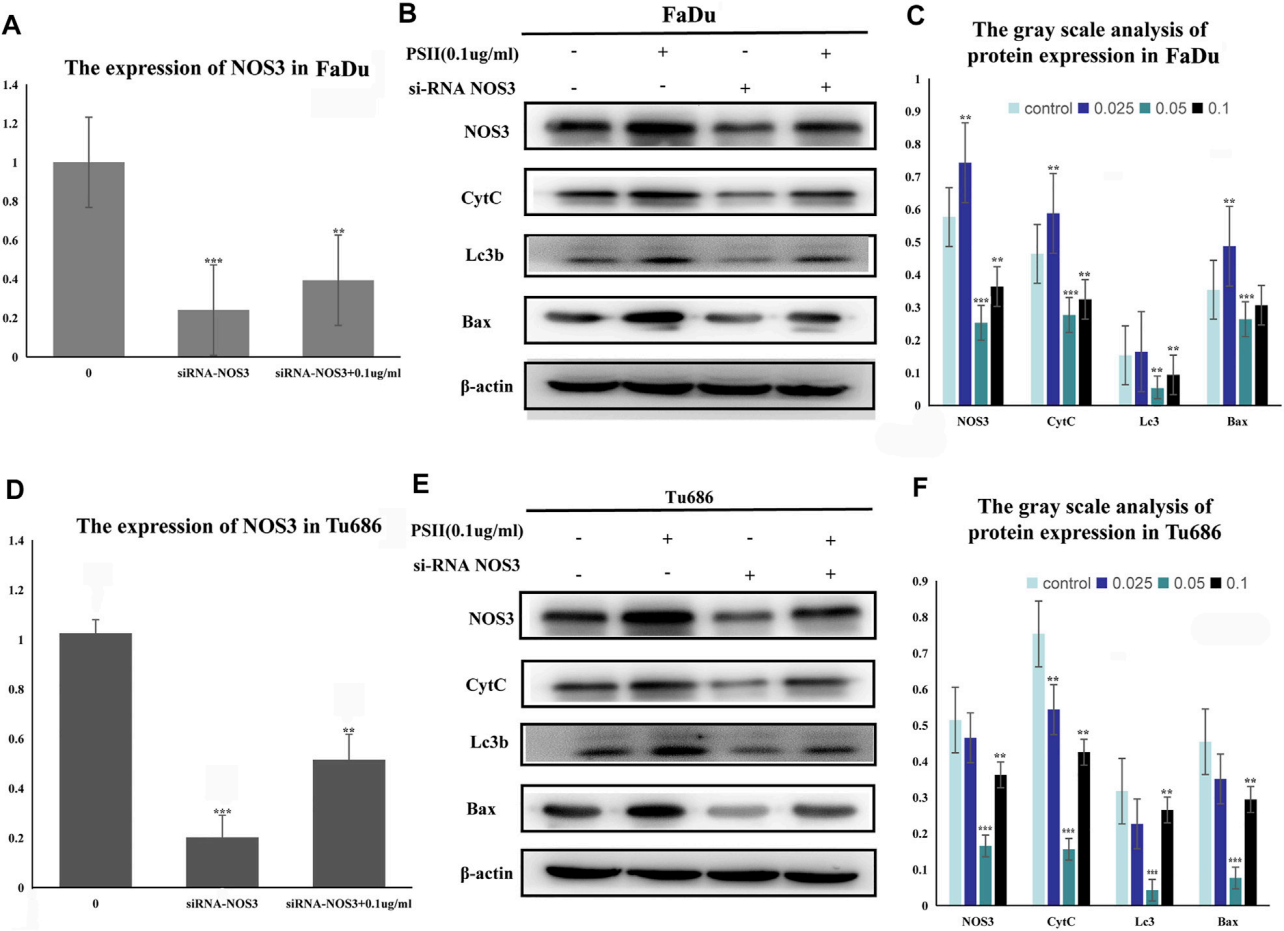
Inhibitive effect of PSII on human squamous cell carcinoma cells by targeting NOS3. **(A)** The mRNA expression of NOS3 in FaDu cells by RT-PCR assay after transfection. **(B)** The transfection of NOS3 siRNA reversed the protein expression induced by PSII (NOS3, CytC, Lc3b, and Bax) in FaDu cells. **(C)** The gray scale analysis of protein expression in FaDu cells by Western blot. **(D)** The mRNA expression of NOS3 in Tu686 cells by RT-PCR assay after transfection. **(E)** The transfection of NOS3 siRNA reversed the protein expression induced by PSII (NOS3, CytC, Lc3b, and Bax) in Tu686 cells. **(F)** The gray scale analysis of protein expression in Tu686 cells by Western blot. We used β-actin as control. All the experiments were carried out three times. Each data represent the mean ± SD of three independent experiments (*p < 0.05; **p < 0.01; ***p < 0.001 vs the control group).

We studied the inhibitory effect of PSII in an *in vivo* mouse tumor, which we developed by injecting Tu686 HNSCC cells into both sides of the flank of BALB nude mice. As shown in Figures 9A,B, the volume of xenograft tumor growth was significantly reduced after PSII treatment compared with the control group. On the 60th day, as shown in Figure 9B, the average volume of tumors in the PSII group (46.89 ± 3.99 mm^3^) was smaller than that in the control groups (273.71 ± 10.07 mm^3^), and there was no statistical difference in weight between the two groups. Our analysis of the protein levels of NOS3, Bcl-2, and Ki67 by IHC staining indicated, as shown in Figure 9C, that treatment with PSII led to decreased protein levels of Bcl-2 and Ki67 and an increased protein level of NOS3. Furthermore, the histopathological examination showed that PSII had no adverse effects on the lung, heart, spleen, liver, and kidney. Additionally, the Western blot analysis of mouse tumors showed that the inhibitory effect of PSII on the growth of subcutaneous tumors is mediated through the mitochondrial pathway, cell autophagy, and apoptosis. All the *in vivo* results indicated that PSII induced autophagy and apoptosis in HNSCC cells.

## Discussion

In 2018, there were more than 5,80,000 newly diagnosed cases of HNSCC. However, despite advances in therapeutic approaches, survival rates remain poor for patients with HNSCC (Jung et al., 2020; Kitamura et al., 2020). More than half of HNSCC patients suffer relapse, and most die from distant metastasis, and lymph nodes metastases play an important role (Shah et al., 2020; Wang et al., 2020). Therefore, it is important to fully understand the molecular signaling pathways that contribute to HNSCC proliferation and metastasis in order to develop more effective therapeutic methods.

For thousands of years, Paridis saponin (PS) and its components have always been used as hemostatic regulatory, antifungal, anti-inflammatory, antimicrobial, and antibacterial medications in China (White et al., 2015; Moldogazieva et al., 2018). Recent studies have reported promising roles of PS in the regulation of glycolytic and lipid metabolism (Man et al., 2017; Man et al., 2020). In addition, the PSII extract has been approved as an antitumor agent for many years in China, but its mechanism of action is not well understood. In our research, we investigated the therapeutic effects of PSII on various HNSCC cells and showed that PSII can inhibit the viability and motility of HNSCC cell lines (Figures 2–4), cancer stem cells (Figure 5), and primary cultured fibroblasts (Figure 6).

**FIGURE 10:**
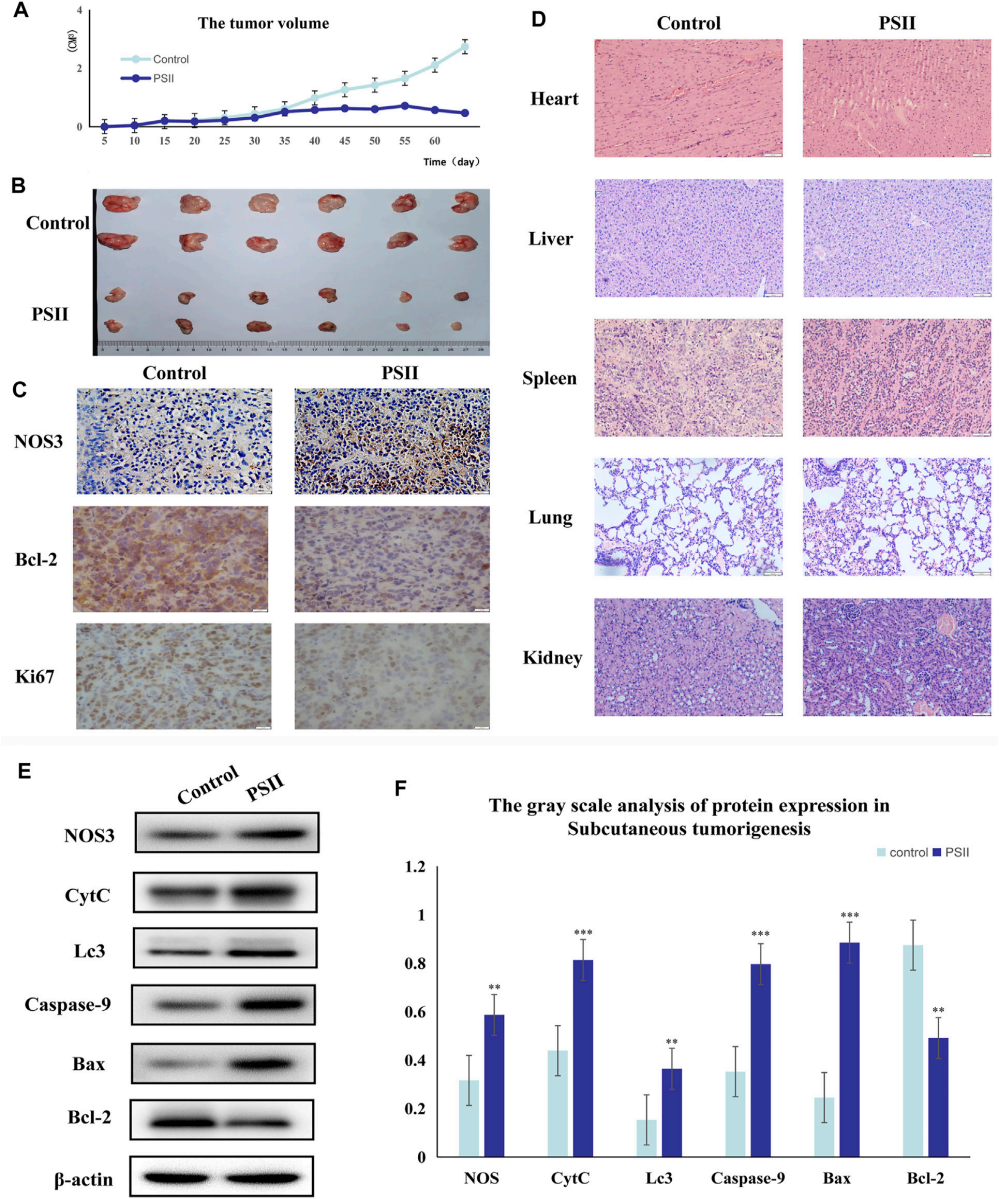
Suppression of tumorigenesis in a xenograft model. **(A)** Growth curves of the volumes of xenograft tumors after the injection of Tu686 cells. **(B)** The representative tumor pictures. **(C)** IHC analysis of NOS3, Bcl-2, and Ki67. **(D)** Histopathological examination of the lung, heart, kidney, liver, and spleen. **(E)** The protein expression of tumorigenesis in a xenograft model by Western blot analysis. **(F)** The gray scale analysis of protein expression in Tu686 cells by Western blot. Bars, 50 µm. All the experiments were carried out three times. The bar graphs and the table show quantification of the results, and each data represent the mean ± SD of three independent experiments (*p < 0.05; **p < 0.01; ***p < 0.001 vs the control group).

Sixty years ago, researchers found that cancer cells could reprogram their metabolic capability to develop metastastic potential. Metabolism plays an increasingly important role in cancer development and has become a new research field. From the research on metabolism, researchers have found promising approaches for development of cancer therapies (Lee et al., 2012; Zuo and Wan, 2019). In order to detect the metabolites affected by PSII, we performed untargeted metabolomics analyses of three HNSCC cell lines with/without PSII. The metabolites in the samples were detected by UHPLC-Q-TOF-MS. The retention time and molecular weight of the metabolites in the samples were compared with those in the local database. From the proportion of various metabolites shown in Figures 10C,D, we were able to find some key differentially expressed metabolic enzymes, such as hexokinases (HKs), phosphoglycerate kinase (PGK), pyruvate kinases (PK), and lactate dehydrogenase (LDH), which have been found to be closely related to tumor aerobic glycolysis (Zhang et al., 2018; Shi et al., 2019; Yeung et al., 2019). In addition, metabolites in the PSII-treated group showed significant differences compared with those in the control group.

Additionally, we predicted a downstream target of PSII in cancer using the Chinese Medicine Data Website (http://herb.ac.cn/), which is a high-throughput experiment- and reference-guided database of TCM compounds. From this website, we identified potential targets of PSII, such as AHSA1, NOS3, IKBKB, and BCL2. Cross-analysis with metabolomics results revealed that the NOS3-mediated energy metabolism pathway may be the pathway mediating the effects of PSII. Endothelial nitric oxide synthase (NOS3) is an important member of the nitric oxide synthase (NOS) enzyme family that plays an important role in nitric oxide (NO) production. The biological function of NOS enzymes and the activity of NO have been the focus of cancer research for many years. NO metabolism plays an important role in various stages of tumor development, such as in oncogene activation, inhibition of DNA repair enzymes, DNA damage, and tumor suppressor genes, as well as the regulation of apoptosis and metastasis. For many years, the dual role of NO in cancer has been recognized, and more and more studies are investigating deeper mechanisms. The inhibition of oxidative phosphorylation in tumor cells was found to lead to elevated glycolytic metabolism (Ghashghaeinia et al., 2019; Najjar et al., 2019). Compared with their differentiated offspring, stem cells rely more on glycolysis which preferentially metabolizes lactate via mitochondrial respiration (Deshmukh et al., 2016; Liang et al., 2020), indicating that the changes occurring in metabolism occur earlier than those in stemness (Shyh-Chang et al., 2013). In recent years, NOS3 has been found to play various roles in malignant tumors. For instance, NOS3 is closely related to oxidative stress, autophagy, and energy metabolism. In our project, we examined the effects of NOS3 with siRNA targeting its expression in HNSCC cells. Moreover, the Western blot analysis indicated that the expression of Bax, CytC, and Lc3b as well as partially could be reversed in FaDu and Tu686 cells (Figures 8C,D). These results confirmed that NOS3 was a downstream target of the PSII extract treatment in HNSCC. Also, as shown in Figure 7, the mRNA and proteins expression levels of genes involved in the mitochondrial pathway (CytC), cell autophagy (Lc3b), and apoptosis (Bax, Bcl-2, and caspase-9) were all changed after PSII treatment. In order to determine the curative effect of PSII *in vivo*, Tu686 HNSCC cells were subcutaneously injected into the nude mice. The volume of xenograft tumor was suppressed after PSII treatment as shown in Figure 9. To examine the mechanism through which PSII exerts its effects *in vivo*, we measured NOS3, Bcl-2, and Ki67 protein expression by immunohistochemical staining. In addition, the histopathological examination showed that PSII had no adverse effects on the heart, liver, spleen, lung, and kidney. These results suggested that PSII extract induced autophagy and apoptosis in HNSCC cells *in vivo* with no side effects on organs.

As mentioned earlier, the TME, which includes CSCs, fibroblasts, metabolites, and various kinds of immune cells (Reina-Campos et al., 2017; García-Cañaveras et al., 2019; Li et al., 2019), can affect many steps of tumor development, including immune escape, distant metastasis, drug resistance, and recurrence (Batlle and Clevers, 2017; Jahanafrooz et al., 2020). Redox signaling and oxidative stress are major components of the TME that could regulate various kinds of metabolites (Catalano et al., 2013; Moldogazieva et al., 2018; Bonanno et al., 2019). Mitochondria are both generators and targets of ROS, so oxidative stress plays an important role in mitochondrial dysfunction. Mitochondrial turnover is dependent on autophagy, which declines with age and is frequently dysfunctional in cancer (White et al., 2015). Autophagy is also necessary to recycle ferritin, and autophagy defects cause perturbation in iron homeostasis that increases susceptibility to oxidative stress. The crosstalk between autophagy, redox signaling, and mitochondrial dysfunction is not well understood (Lee et al., 2012).

In this work, we reported that PSII could inhibit the viability and motility of HNSCC cell lines, cancer stem cells, and primary cultured fibroblasts from the tissue through the mitochondrial pathway by targeting NOS3 as well as inducing autophagy and apoptosis through a series of changes of metabolites *in vivo* and *in vitro*. Our study indicated that PSII may be a novel strategy for HNSCC, and the metabonomics method can be a novel tool to investigate and establish the antitumor effects of TCM preparations.

## Data Availability

The original contributions presented in the study are included in the article/supplementary files; further inquiries can be directed to the corresponding author.
